# Multi-Organ Omics-Based Prediction for Adaptive Radiation Therapy Eligibility in Nasopharyngeal Carcinoma Patients Undergoing Concurrent Chemoradiotherapy

**DOI:** 10.3389/fonc.2021.792024

**Published:** 2022-01-31

**Authors:** Sai-Kit Lam, Yuanpeng Zhang, Jiang Zhang, Bing Li, Jia-Chen Sun, Carol Yee-Tung Liu, Pak-Hei Chou, Xinzhi Teng, Zong-Rui Ma, Rui-Yan Ni, Ta Zhou, Tao Peng, Hao-Nan Xiao, Tian Li, Ge Ren, Andy Lai-Yin Cheung, Francis Kar-Ho Lee, Celia Wai-Yi Yip, Kwok-Hung Au, Victor Ho-Fun Lee, Amy Tien-Yee Chang, Lawrence Wing-Chi Chan, Jing Cai

**Affiliations:** ^1^ Department of Health Technology and Informatics, The Hong Kong Polytechnic University, Hong Kong, Hong Kong SAR, China; ^2^ Department of Clinical Oncology, Queen Mary Hospital, Hong Kong, Hong Kong SAR, China; ^3^ Department of Clinical Oncology, Queen Elizabeth Hospital, Hong Kong, Hong Kong SAR, China; ^4^ Department of Clinical Oncology, The University of Hong Kong5Comprehensive Oncology Centre, Hong Kong Sanatorium & Hospital, Hong Kong, Hong Kong SAR, China; ^5^ Comprehensive Oncology Centre, Hong Kong Sanatorium & Hospital, Hong Kong, Hong Kong SAR, China

**Keywords:** nasopharyngeal carcinoma, adaptive radiotherapy, radiomics, dosiomics, multiomics approach

## Abstract

**Purpose:**

To investigate the role of different multi-organ omics-based prediction models for pre-treatment prediction of Adaptive Radiotherapy (ART) eligibility in patients with nasopharyngeal carcinoma (NPC).

**Methods and Materials:**

Pre-treatment contrast-enhanced computed tomographic and magnetic resonance images, radiotherapy dose and contour data of 135 NPC patients treated at Hong Kong Queen Elizabeth Hospital were retrospectively analyzed for extraction of multi-omics features, namely Radiomics (R), Morphology (M), Dosiomics (D), and Contouromics (C), from a total of eight organ structures. During model development, patient cohort was divided into a training set and a hold-out test set in a ratio of 7 to 3 *via* 20 iterations. Four single-omics models (R, M, D, C) and four multi-omics models (RD, RC, RM, RMDC) were developed on the training data using Ridge and Multi-Kernel Learning (MKL) algorithm, respectively, under 10-fold cross validation, and evaluated on hold-out test data using average area under the receiver-operator-characteristics curve (AUC). The best-performing single-omics model was first determined by comparing the AUC distribution across the 20 iterations among the four single-omics models using two-sided student *t*-test, which was then retrained using MKL algorithm for a fair comparison with the four multi-omics models.

**Results:**

The R model significantly outperformed all other three single-omics models (all p-value<0.0001), achieving an average AUC of 0.942 (95%CI: 0.938-0.946) and 0.918 (95%CI: 0.903-0.933) in training and hold-out test set, respectively. When trained with MKL, the R model (R_MKL) yielded an increased AUC of 0.984 (95%CI: 0.981-0.988) and 0.927 (95%CI: 0.905-0.948) in training and hold-out test set respectively, while demonstrating no significant difference as compared to all studied multi-omics models in the hold-out test sets. Intriguingly, Radiomic features accounted for the majority of the final selected features, ranging from 64% to 94%, in all the studied multi-omics models.

**Conclusions:**

Among all the studied models, the Radiomic model was found to play a dominant role for ART eligibility in NPC patients, and Radiomic features accounted for the largest proportion of features in all the multi-omics models.

## Introduction

Nasopharyngeal carcinoma (NPC) presents immediate proximity to a variety of surrounding critical healthy organs such as spinal cord and brainstem within an intricated nose-pharynx ministry, dysfunction of which can incur catastrophic complications. At present, concurrent chemo-radiotherapy (CCRT) is a standard-of-care remedy for advanced NPC patients; adoption of Intensity-modulated Radiotherapy (IMRT) allows for highly conformal and precise dose delivery to the treatment targets, meanwhile protecting the adjacent healthy tissues. Notably, the success of treatment relies on an assumption that the patient anatomy remains throughout the 6-7 weeks of IMRT course. In response to treatment perturbations, however, tumors and surrounding healthy organs may exhibit significant morphometric volume and/or geometric alterations, which may jointly alter patient anatomy and jeopardize the efficacy of the original treatment plan (1–3). The issue of these variabilities can be more detrimental in the IMRT era, where slight anatomic deviations may deleteriously lead to significant dosimetric consequences due to the sharp dose falloff beyond the target lesions. Confronted with this, Adaptive Radiotherapy (ART), a modification of the original treatment plan, has been introduced to compensate for these patient-specific variations. The dosimetric and clinical benefits of ART for NPC patients have been well-documented in the literature (1–7).

Notwithstanding, ART generally involves re-imaging, re-segmentations of tumor and organs-at-risk (OARs), and re-planning, requiring a highly specialized multidisciplinary team. This labor-intensive and time-consuming nature of ART procedures preclude the feasibility of routine ART practice on a patient basis in clinic. In light of this, tremendous effort has been constantly made to evaluate the underlying morphometric and geometric variations of patient anatomy amid the radiotherapy course, in the hope of streamlining clinical implementation of ART (8–20).

Radiation dose has long been regarded as a prime attribute for morphometric volume change of tumors, neck lesions and bilateral parotid glands throughout the treatment course. Bahl et al. (8) prospectively analyzed volumetric alterations in 20 NPC patients between pre-treatment computed tomography (CT) and mid-treatment CT at the 17^th^ fraction. They reported approximately 30% shrinkage of high-risk gross-tumor-volume (GTV), which was accompanied with a significantly increased median dose of 7.2-7.7 Gy to and reduced volume of bilateral parotid glands. Another prospective study by Cheng et al. (9) demonstrated that the anatomic tissue shrinkage was dependent on radiation dose received. They analyzed repeated planning CT and magnetic resonance images (MRI) at 30-Gy and 50-Gy intervals and reported that the shrinkage of both primary NPC tumor and nodal lesions against pre-treatment baselines were higher when 50-Gy was delivered (13% and 29%, respectively) than that when 30-Gy was given (9% and 16%, respectively) and a similar trend was also observed for bilateral parotid glands. Further evidence was also observed by Hu et al. (10) who analyzed 40 re-planned NPC patients and confirmed the significant shrinkage of 35% in clinical-target-volume, and by Murat et al. (11) who reported a median reduction of 27% and 43% in primary and nodal GTV, respectively, in 48 re-planned head-and-neck cancer patients.

Notably, volumetric shrinkages of these organ structures are often accompanied with geometric shifts of internal structures (12, 13) and/or body contour modification (14, 15), which may in concert contribute to an elevated risk of ill-fitted immobilization cast during daily setup (14, 15) and/or detrimental consequences following treatment [e.g., overdosing to OARs (7, 16, 17), underdosing to targets (7, 12)], triggering the demand for ART. In view of this, research community has introduced numerous criteria as ART triggers (11, 12, 18–20), mainly on dosimetric aspects. Nevertheless, most of these factors require close monitoring throughout the radiotherapy course for each patient, pre-treatment prediction of ART eligibility is greatly demanding. Further, these factors are deficient in capturing inter-patient disparity in intrinsic biologic response of tissue upon receiving treatment perturbation.

Until more recently, emerging Radiomics has opened up opportunities for divulging concealed biologic traits and genetic association of tumor and organ structures (21–23). There is mounting evidence in the literature showing the power of Radiomics in predicting treatment response on the ground of volume shrinkage in various cancer diseases (24–29), which has laid great foundation for Radiomics prediction of ART demand in cancer patients. Ramella et al. performed radiomic analysis on pre-treatment CT images of replanned non-small cell lung cancer patients and generated a radiomic signature for prediction of tumor shrinkage during chemo-radiotherapy, yielding an Area Under the Receiver Characteristics Curves (AUC) of 0.82 (27). For the first time, Yu et al. generated several radiomic models for ART eligibility in NPC patients using tumoral radiomic features from multi-parametric pre-treatment MRI, achieving AUCs ranging from 0.75 to 0.93 (15). It is worth noting that ART eligibility is multifactorial in nature. Joint response of multiple organ structures upon treatment perturbations, treatment aggressiveness, and pre-treatment geometric and morphologic condition of patient anatomy, may all come into play for triggering ART.

Therefore, it is pertinent to investigate the role of these attributes, in the form of -omics features, from multiple relevant organ structures within head-and-neck regions using pre-treatment CT, MRI, contours, and three-dimensional dose map for prediction of ART eligibility in NPC patients, which constituted the main objective of this present study. The success of this study may provide the community with valuable insights into developing ART screening strategies in future, particularly in view of the soaring demand of ART in this vulnerable subgroup of cancer sufferers in the IMRT era.

## Methods and Materials

### Patient Data

This study is a retrospective analysis of 261 NPC patients who received radiotherapy at Hong Kong Queen Elizabeth Hospital between 2012 and 2015. Patient informed consent was waived due to the retrospective nature of this study. Patients were included if they (1) were diagnosed with biopsy-proven primary NPC without presence of distant metastasis and co-existing tumors of other types at presentation (2), underwent curative concurrent chemo-RT (CCRT) or CCRT plus adjuvant chemotherapy (AC), and (3) were treated with Helical Tomotherapy. Patients were excluded if they (1) received induction chemotherapy before CCRT treatment, or (2) received RT-alone without concurrent chemotherapy, or (3) did not receive injection of contrast agent for obtaining planning contrast-enhanced CT (CECT) images or planning contrast-enhanced T1-w (CET1-w) MR images, or (4) did not have complete set of clinical/image data. The binary status of whether or not an individual patient has undergone ART treatment during their main course of RT at the discretion of radiation oncologist was chosen as the clinical endpoint for this study. Patients were labelled as 1 if he/she has received ART treatment, otherwise were labelled as 0.

### Image Acquisition

All the enrolled patients underwent pre-treatment planning CECT and MRI scans, which were retrospectively retrieved in Digital Imaging and Communications in Medicine (DICOM) format, archived using Picture Archiving and Communication System (PACs). Details of imaging parameters can be found in **Supplementary A1**.

### Volume-of-Interest (VOI) Definition

**Figure 1:**
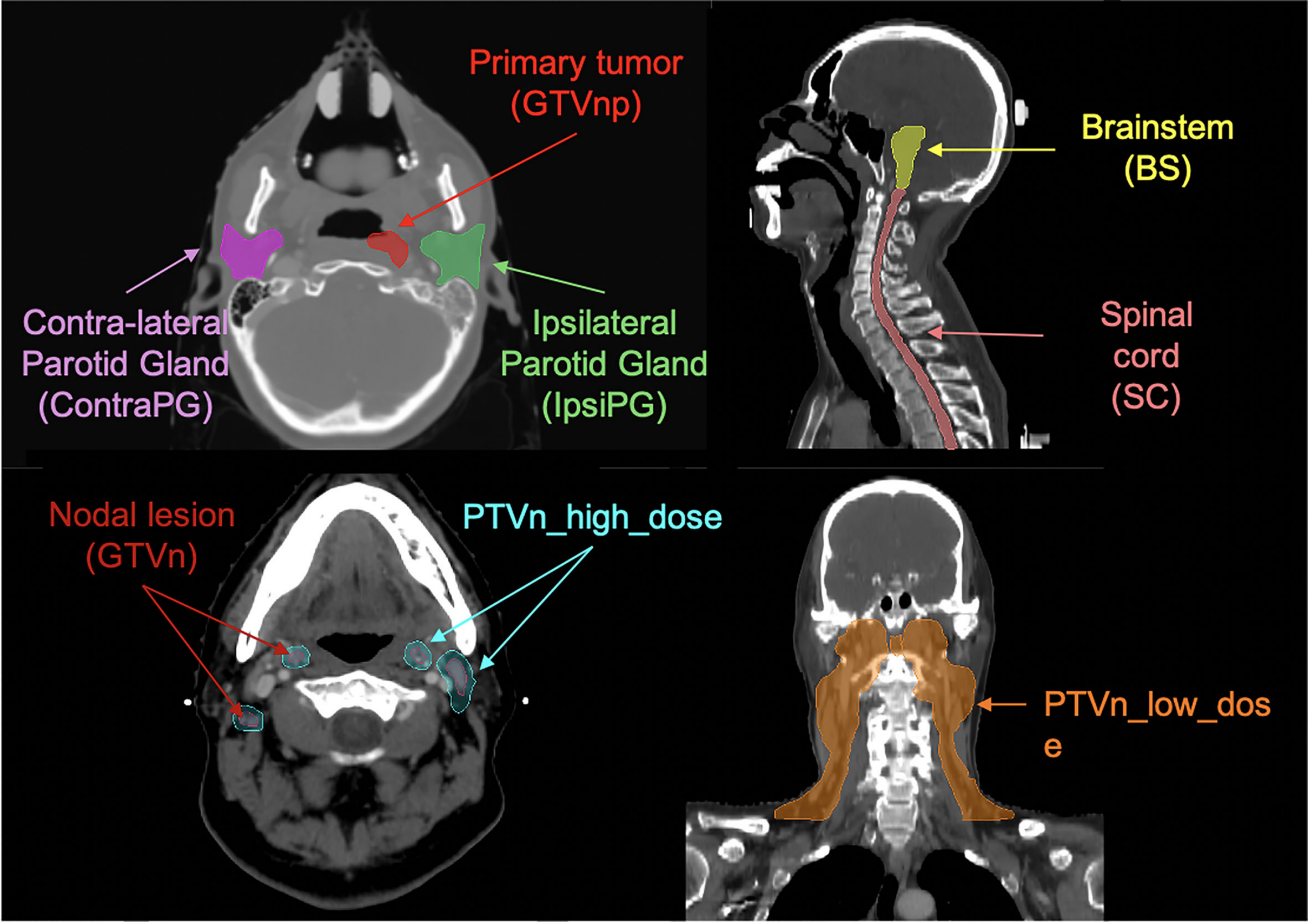
Illustration of the eight VOIs involved in this study.

There were a total of 8 different VOIs of organ structures involved in this study, including gross-tumor-volume of primary NPC tumor (GTVnp) and metastatic lymph nodes (GTVn), ipsi-lateral parotid gland (IpsiPG), contra-lateral parotid gland (ContraPG), brainstem (BS), spinal cord (SC), high-dose and low-dose regions of nodal planning target volume (PTVn_high_dose for the PTVn with the prescribed dose level of 70-Gy, PTVn_low_dose for the PTVn with the prescribed dose level of 60-Gy, respectively). **Figure 1** illustrates location of each VOI involved in this study.

GTVnp was manually delineated on axial CT slices after registration with planning MR images, and GTVn was delineated on CECT images by an experienced radiation oncologist specializing in head-and-neck cancers with accreditations, in accordance with International Consensus Guidelines for the CT-based delineation of neck levels (30). To determine whether each of the segmented parotid glands (PG) belongs to IpsiPG or ContraPG for each patient, the minimum geometric distance between a particular voxel point on the PG volume and all voxel points on the GTVnp surface was first determined. This procedure was repeated for another voxel point on the PG volume until the minimum distances between each of all the voxel points on the PG volume and the GTVnp surface were determined. Lastly, a median value of these calculated minimum distances was obtained to determine the overall proximity of that PG to the GTVnp for each patient. The PG with smaller median value of the minimum distances was denoted as IpsiPG, otherwise it was denoted as ContraPG. All segmentations were carried out using Varian ARIA and Eclipse treatment planning system v13 (Varian Medical Systems Inc, Palo Alto, CA).

### Multi-Omics Feature Extraction

#### Radiomics (R) and Morphology (M)

Prior to radiomic feature extraction, a series of image preprocessing steps were performed on CECT and MR images according to well-recognized recommendations from the Image Biomarker Standardisation Initiative (IBSI) guidelines (31), using our in-house developed Python-based (v3.7.3) platform. Details of the image preprocessing procedures can be found in **Supplementary A2**.

In this study, 4 different VOIs of organ structures (GTVnp, GTVn, IpsiPG and ContraPG) were involved in radiomic feature calculations. Extraction of radiomic features was performed using the publicly available SimpleITK (v1.2.4) and PyRadiomics (v2.2.0) packages embedded in our platform in accordance with the IBSI guidelines (31). Radiomic features can be generally divided into three major families: morphologic features, first-order statistics, and texture features which can be further categorized into Gray Level Difference Matrix (GLDM), Gray Level Cooccurrence Matrix (GLCM), Gray Level Run Length Matrix (GLRLM), Gray Level Size Zone Matrix (GLSZM), Neighboring Gray Tone Difference Matrix (NGTDM) classes. Radiomic feature calculations were performed on CECT, CET1-w and T2-w MR images, with and without being filtered by Laplacian of Gaussian (LoG) filter (kernel size: 1-mm, 3-mm, 6-mm) and wavelet filters (HHH, HLL, LHL, LLH, LHH, HLH, HHL, LLL). In this study, morphologic features of all the 4 VOIs were separated from the radiomic feature set, resulting in a total of 6,348 radiomic features for each studied VOI. A total of 14 morphologic features, including elongation, flatness, least axis length, major axis length, minor axis length, maximum 2D diameter column, maximum 2D diameter row, maximum 2D diameter slice, maximum 3D diameter, mesh volume, sphericity, surface area, surface volume ratio, voxel volume, for each of the 4 VOIs (i.e., GTVnp, GTVn, IpsiPG, and ContraPG) were combined to form a set of 56 features. Detailed definitions of the radiomic and morphologic features can be found on the Pyradiomics documentation (https://pyradiomics.readthedocs.io/en/latest/features.html).

#### Dosiomics (D)

All the 8 different VOIs of organ structures were employed for dosiomic feature calculation using RT dose data. Conventional dose-volume histogram (DVH) does not contain information on spatial dose distribution within irradiated organs. By contrast, dosiomics is capable of characterizing spatial pattern of local radiation dose distributions within the 8 studied VOIs. It has been extensively studied in various predictive modelling for cancer prognosis and treatment responses (32, 33). In this study, dosiomic features of DVH curve points for the 8 VOIs were calculated based on the method adopted by Gabryś et al. (34), examples include but not limited to maximum dose, minimum dose, mean dose, volume of the VOI receiving at least certain dose levels, and minimum dose received by certain volume of the VOI. Besides, spatial dose distribution within each studied VOI was extracted to comprehensively depict the heterogeneity of deposited dose, such as dose gradients along the three imaging axes (x-, y- and z-directions). The definitions of these features were described in a previous publication by Buettner et al. (35). Further, the three-dimensional (3D) dose distribution within each studied VOI was transformed into a 3D image, such that radiomics-alike dosiomics features were subsequently calculated using the PyRadiomics package; examples include first-order dose statistics, GLDM, GLCM, GLRLM, GLSZM and NGTDM. A total of 1608 dosiomic features were extracted from the 8 VOIs in this study.

#### Contouromics (C)

In this work, we extracted features that depict complex geometric relationships between 4 pairs of VOIs of organ structures (GTVnp and IpsiPG, GTVnp and ContraPG, GTVnp and SC, and PTVn_low_dose and SC), on the ground that the implementation of ART is triggered by change of geometric relationship of different internal organs within head and neck regions. These features were extracted from the RT contour data. For the first time, they were termed as “Contouromics” in this study. For each of the VOI pairs, a series of contouromic features were calculated from a distance descriptor overlap-volume histogram (OVH), as adopted in a previous publication (36); for instance, the maximum and minimum distances between SC and PTVn_low_dose during the treatment planning stage were calculated as the distances on the OVH at zero and full volume, respectively. In this study, the calculation of OVH was implemented using the algorithm employed in a previous publication (37). Besides, an angle descriptor projection-overlap-volume (POV), defined as one VOI that overlaps with the parallel projection of another VOI at specific projection angle, was used for further divulging potential contouromic features from the VOI pairs. A total of 132 contouromic features were extracted from the 4 pairs of VOIs in this study. **Table 1** summarizes the sources of VOIs involved in calculation of the four types of -omics features studied. 

**Table 1:** Summarizes the sources of VOIs involved in calculation of the four types of -omics features studied.

Radiomics (R)	Morphology (M)	Dosiomics (D)	Contouromics (C)
CECT-GTVnp	GTVnp	GTVnp	PTVn_low_dose-SC
CECT-GTVn	GTVn	GTVn	GTVnp-IpsiPG
CECT-IpsiPG	IpsiPG	IpsiPG	GTVnp-ContraPG
CECT-ContraPG	ContraPG	ContraPG	GTVnp-SC
CET1w-GTVnp		BS	
CET1w-IpsiPG		SC	
CET1w-ContraPG		PTVn_high_dose	
T2w-GTVnp		PTVn_low_dose	
T2w-IpsiPG			
T2w-ContraPG			

### Determination of Optimal Feature Selection (FS) Algorithms for Each -Omics Dataset

Feature dimensionality reduction is considered essential in machine learning when it comes to minimizing the risk of model overfitting. Although there are a multitude of unsupervised and supervised FS algorithms currently available for assessing redundancy and outcome relevance of the studied features, an optimal combination of both kinds of FS algorithms remains unclear. In this study, a total of 6 unsupervised and 4 supervised FS algorithms that have been commonly adopted in machine learning were studied (38) and are publicly available (https://jundongl.github.io/scikit-feature/algorithms.html), giving rise to a resultant amount of 24 FS combinations **(** **Supplementary Figure S1** **).**

A proper selection of FS combination for a particular feature set is crucial to ensure that the final selected features of a prediction model are of high discriminability (i.e., high score of Area Under the Receiver Operating Characteristics Curve, AUC score) and high reproducibility under multiple train/test splits of the dataset (i.e., high feature output stability score). To this end, we adopted a strategic workflow **(** **Supplementary Figure S2** **)** to calculate both scores and determined the optimal FS combination using a decision graph **(** **Supplementary Figure S3** **)** for a particular -omics dataset. More details can be found in **Supplementary A3**.

### Development and Evaluation of ART Prediction Models

In this study, a total of 4 single-omics models (R, M, D, C) and 4 multi-omics models (RM from R+M, RD from R+D, RC from R+C, RMDC from R+M+D+C) were developed using the corresponding -omics features from multiple VOIs of organ structures.

**Figure 2:**
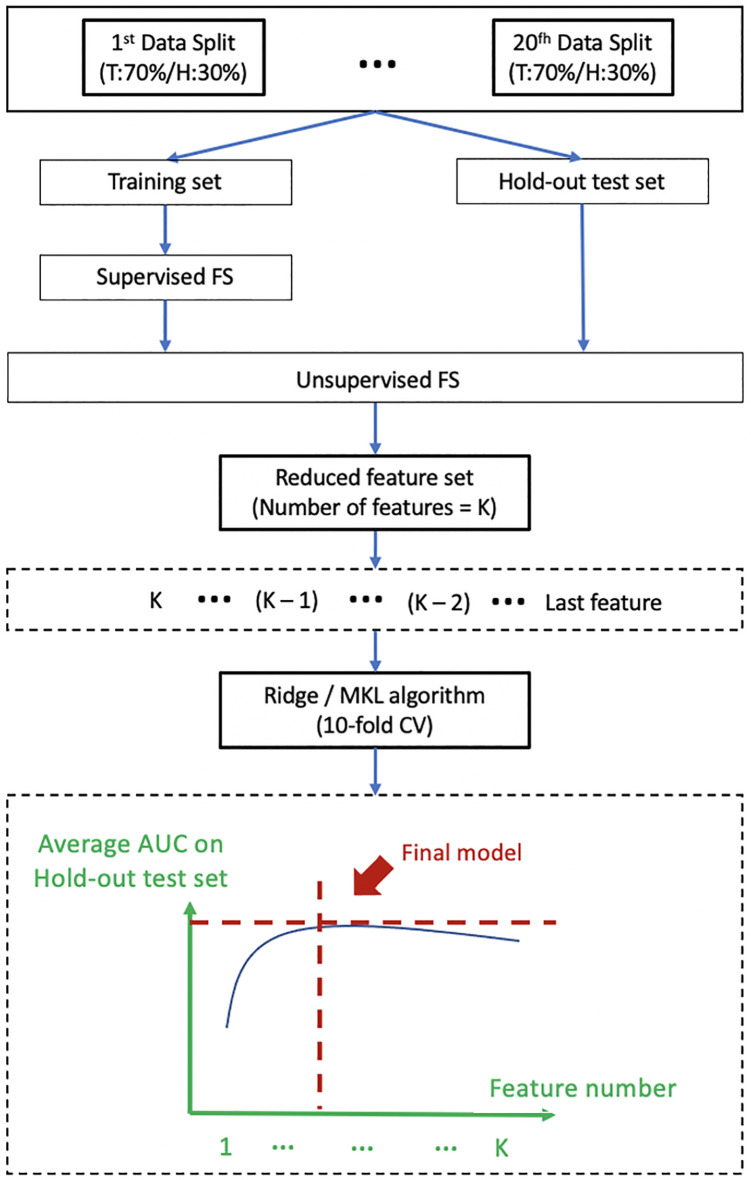
Shows a schematic diagram for model development. T, Training set; H, Hold-out test set; FS, feature selection; MKL, Multi-Kernel Learning; CV, Cross-Validation; AUC, Area Under the Receiver Operating Characteristics Curves.

**Figure 2** shows a schematic diagram for model development. The patient cohort was divided into a training dataset and a hold-out test dataset in a ratio of 7 to 3 *via* 20 iterations. The optimal supervised FS algorithm was applied only to the training dataset of each iteration to maintain clinical relevance of the remnant features. The optimal unsupervised FS algorithm was subsequently applied to remove highly redundant features, leading to a reduced feature set of K features. Development of prediction models was conducted with the initial K features using the Ridge algorithm (for single-omics model) or Multi-Kernel Learning (MKL) algorithm (for multi-omics model) *via* a 10-fold cross-validation (CV) within the training set to mitigate the risk of model overfitting. Evaluation of model discriminability, in aspects of AUC, was performed on the hold-out test set of each iteration. The model development process was repeated on (K-1) features after removing the feature of the lowest ranking of frequency of occurrence across the 20 iterations until one feature remained in the feature set. An optimal prediction model was finally determined when the average AUC on the hold-out test datasets reached its maximum.

With regard to the model training algorithm, Ridge classifier was adopted for generation of the 4 single-omics models. It is a typical statistical approach for resolving bias-variance trade-off with the use of a linear function; the principles and advantages of Ridge algorithm have been well-documented (39). On the other hand, MKL algorithm was applied for development of multi-omics models in this study. Unlike single-omics features, different types of multi-omics data may contain distinctly different data representations. Ridge algorithm is deficient in capturing the difference in representations of multi-omics data and non-linear relationship between predictors and prediction outcome. Therefore, MKL was adopted in this study with an attempt to divulging complementary (non-linear) relationship between different types of -omics features and prediction outcomes. Specifically, two types of kernels (Gaussian and Polynomial) with a range of kernel parameters were applied. Each kernel was embedded into the feature space of a given multi-omics feature set for subsequent multi-omics fusion. **Supplementary Figure S4** illuminates the multi-omics fusion framework in our study. More details of the MKL algorithms can be found in **Supplementary A4** and a previous publication (40).

### Model Comparison and Statistical Analysis

For single-omics models, discriminability of the final radiomic model (R), in terms of distribution of the AUC scores across the 20 iterations, was compared against the other 3 single-omics models (M, D, and C) in both training and hold-out test datasets. For multi-omics models, discriminability of the final RMDC model was compared against the other 3 multi-omics models (RM, RD, and RC) in both training and hold-out test datasets. Further, we also compared the best-performing single-omics model against all the 4 studied multi-omics models (RM, RD, RC, and RMDC). With this regard, the selected single-omics model was firstly re-trained using MKL algorithm for achieving a fair comparison with multi-omics models.

Statistical estimates of model discriminability in terms of average AUC, its standard deviation (STD) and 95% confident interval (95%CI) across the 20 iterations for all the studied prediction models were reported in this study. Two-sided paired student *t*-test was employed for the abovementioned comparisons. On the other hand, Chi-square test was employed to assess statistical difference of categorical patient clinical factors between patients who received ART and those who did not, while two-sided student *t*-test was applied for continuous clinical factors. A p-value of ≤ 0.05 was considered statistically significant.

## Results

### Patient Characteristics

A total of 135 NPC patients (35 experienced ART, approximately 26%) were finally considered eligible for this study. **Table 2** summarizes major characteristics of the patients. There were no statistically significant differences in the studied clinical factors between patients who experienced ART and those who did not.

**Table 2:** Patient clinical characteristics.

Clinical factor	Data/p-value	
**Age**	p-value = 0.142	
Average, Range	54	27 - 81
**Gender**	p-value = 0.348	
Male (no.,%)	101	75
Female (no.,%)	34	25
WHO Histologic subtype*	p-value = 0.544	
Type-1 (no., %)	4	3
Type-2 (no., %)	3	2
Type-3 (no., %)	128	95
**T-Stage**	p-value = 0.133	
T1 (no., %)	9	7
T2 (no., %)	9	7
T3 (no., %)	94	70
T4 (no., %)	23	17
**N-Stage**	p-value = 0.146	
N0 (no., %)	1	1
N1 (no., %)	22	16
N2 (no., %)	98	73
N3 (no., %)	14	10
**Overall stage (7^th^ AJCC)**	p-value = 0.077	
Stage-I (no., %)	1	1
Stage-II (no., %)	7	5
Stage-III (no., %)	92	68
Stage-IVA (no., %)	23	17
Stage-IVB (no., %)	12	9
**Initial size of primary tumor (mm^3^)**	p-value = 0.341	
Average, range	43,482	4,537 - 184,333
**Initial size of nodal lesion (mm^3^)**	p-value = 0.202	
Average, range	31,078	501 - 330,143
**Initial total tumor burden** **(primary + nodal lesion) (mm^3^)**	p-value = 0.153	
Average, range	74,560	7,886 - 438,998
**Pre-treatment body weight (kg)**	p-value = 0.265	
Average, range	63	37-102

*WHO histologic subtype of NPC: Type 1: Keratinizing squamous cell carcinoma; Type 2: Non-keratinizing differentiated carcinoma; Type 3: Non-keratinizing undifferentiated carcinoma. AJCC, American Joint Committee on Cancer.

### Optimal FS Combination Determination and Model Development

Optimal combinations of FS algorithms for the 4 single-omics datasets (R, M, D, C) and the 4 multi-omics datasets (RM, RD, RC, RMDC) were determined using the decision graphs **(** **Supplementary Figures S5A–H** **)** and were summarized in **Supplementary Table S1**.

**Supplementary Figures S6A–D** and **S7A–D** illustrate the change of average AUC scores (and its STD shown in shadow) in both training and hold-out test sets against varying number of features for the 4 single-omics models and the 4 multi-omics models, respectively. Final models were determined when the average AUC scores on the hold-out test sets reached its maximum.

**Table 3** summarizes the total number and distribution of the selected features in the final models. Interestingly, it can be observed that radiomic features are dominant in all the four multi-omics models, compared to M, C, and D features.

**Table 3:** A summary of total number and distribution of selected features in the final models.

	Number of Final Selected Features				
	Total	R	M	C	D
**Radiomics (R)**	11	11	*	*	*
**Morphology (M)**	9	*	9	*	*
**Contouromics (C)**	10	*	*	10	*
**Dosiomics (D)**	18	*	*	*	18
**Radiomics (R_MKL)**	23	23	*	*	*
**RM**	33	31	2	*	*
**RC**	28	27	*	1	*
**RD**	38	30	*	*	8
**RDCM**	55	36	3	9	7

*Not applicable.

### Model Comparison

**Figure 3:**
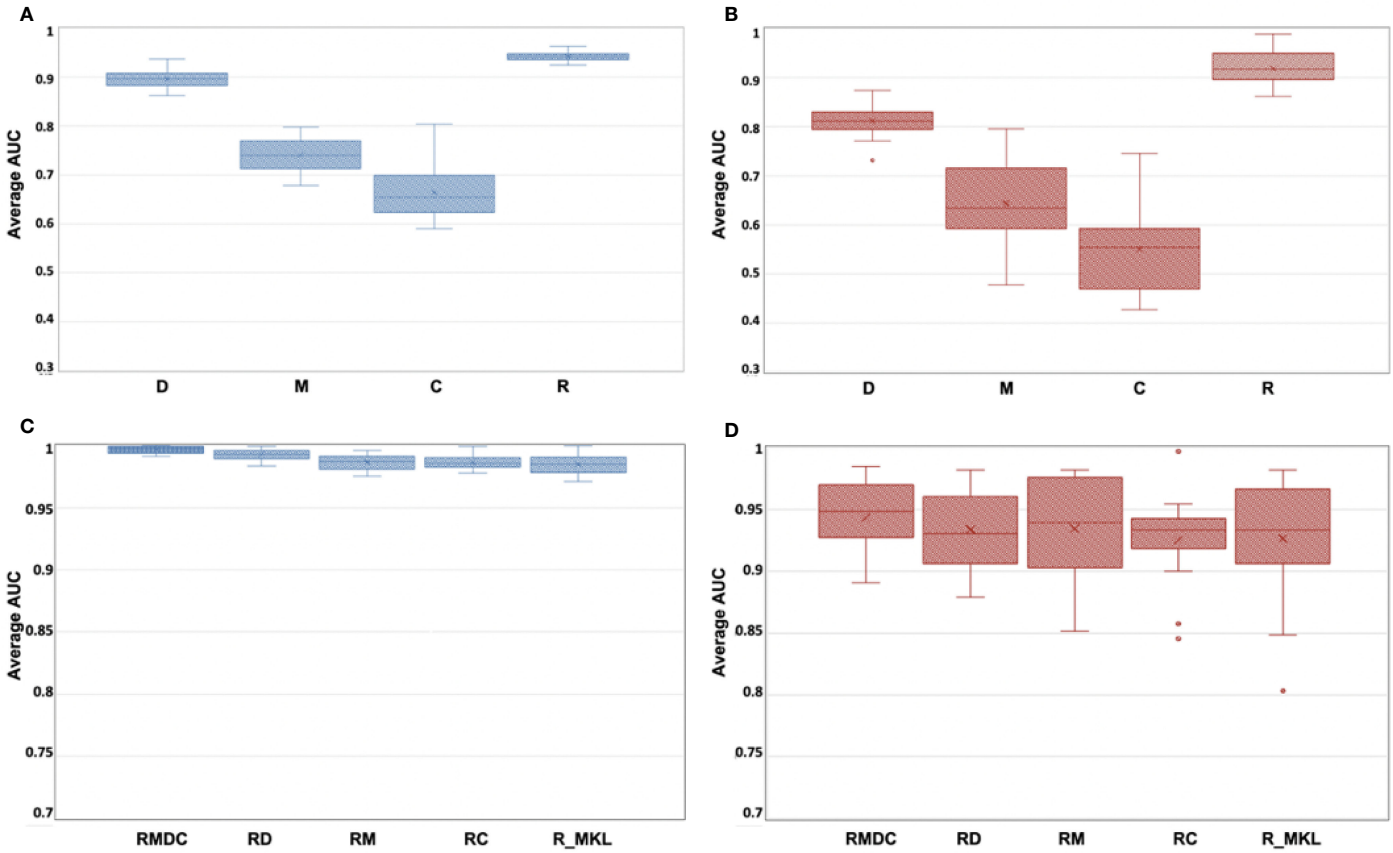
**(A–D)** Box-whisker plots of the average AUC distribution for the final single-omics models in training set **(A)** and hold-out test set **(B)**, and for the multi-omics models and the Radiomic models trained by using MKL algorithms in training **(C)** and hold-out test set **(D)**.

**Figures 3A, B** indicates box-whisker plots of the average AUC distributions for the final single-omics models, and **Figures 3C, D** for the multi-omics models and the Radiomic models trained by using MKL algorithms, in training and hold-out test sets. A summary of the statistical estimates of model performance is provided in **Tables 4A, B**.

**Table 4(A-B):** A summary of statistical estimates on performance of single-omics models (4A), multi-omics models and the Radiomic model trained by using MKL algorithm (4B).

Table 4A	Training Set				Hold-out test set					
	Avg. AUC	STD	95% CI	p-value		Avg. AUC	STD	95% CI	p-value	
Single-omics Model										
Radiomics (R)	0.94	0.01	(0.938,0.946)	Ref		0.92	0.03	(0.903,0.933)	Ref	
Morphology (M)	0.74	0.03	(0.726,0.754)	<0.0001*		0.64	0.08	(0.608,0.677)	<0.0001*	
Contouromics (C)	0.664	0.052	(0.641,0.687)	<0.0001*		0.55	0.082	(0.514,0.586)	<0.0001*	
Dosiomics (D)	0.9	0.02	(0.887,0.903)	<0.0001*		0.81	0.03	(0.798,0.824)	<0.0001*	
**Table 4B**	**Training Set**				**Hold-out test set**					
	**Avg. AUC**	**STD**	**95% CI**	**p-value**		**Avg. AUC**	**STD**	**95% CI**	**p-value**	
Multi-omics Model										
RM	0.99	0.01	(0.983,0.989)	<0.0001*	0.47	0.93	0.04	(0.916,0.952)	0.36	0.62
RD	0.99	0	(0.990,0.994)	<0.01*	<0.001*	0.93	0.03	(0.920,0.947)	0.37	0.64
RC	0.99	0.01	(0.984,0.989)	<0.0001*	0.42	0.93	0.04	(0.909,0.941)	0.14	0.92
RMDC	1	0	(0.995,0.998)	Ref	<0.0001*	0.94	0.03	(0.931,0.956)	Ref	0.21
Radiomic Model (trained by MKL)										
R_MKL	0.98	0.01	(0.981,0.988)	<0.0001*	Ref	0.93	0.05	(0.905,0.948)	0.21	Ref

The symbol (*) represents meeting the level of statistical significance (p < 0.05).

From **Figures 3A, B** and **Table 4A**, it can be seen that the Radiomic model (R) significantly outperformed all other studied single-omics models (p-value < 0.0001), achieving an average AUC of 0.942 (STD: 0.009, 95%CI: 0.938-0.946) in the training set and 0.918 (STD: 0.034, 95%CI: 0.903-0.933) in the hold-out set.

The Dosiomic model (D) was the second best single-omics model with an average AUC of 0.895 (STD: 0.018, 95%CI: 0.887-0.903) in the training set and 0.811 (STD: 0.029, 95%CI: 0.798-0.824) in the hold-out set. This was followed by the Morphologic model (M) which yielded an average AUC of 0.740 (STD: 0.032, 95%CI: 0.726-0.754) in the training set and 0.643 (STD: 0.078, 95%CI: 0.608-0.677) in the hold-out set, while the Contouromic model (C) was the most underperforming model, producing an average AUC of 0.664 (STD: 0.052, 95%CI: 0.641-0.687) in the training set and 0.550 (STD: 0.082, 95%CI: 0.514-0.586) in the hold-out test set.

From **Figures 3C, D** and **Table 4B**, it can be observed that the RMDC model had the highest AUC of 0.997 (STD: 0.003, 95%CI: 0.995-0.998) in the training set and 0.943 (STD: 0.029, 95%CI: 0.931-0.956) in the hold-out set, compared to other types of multi-omics models. While it statistically outperformed the other three studied multi-omics models (RM, RD, and RC) in the training set, it did not reach the statistical significant level in the hold-out test set.

Notably, when the R model was re-trained using MKL algorithm (referred to as R_MKL model), the average AUC boosted to 0.984 (STD: 0.008, 95%CI: 0.981-0.988) in the training set and 0.927 (STD: 0.050, 95%CI: 0.905-0.948) in the hold-out set. The development and performance of the R_MKL model can be seen in **Supplementary Figure S7E**, **Figures 3C, D** and **Table 4B**. Surprisingly, further comparisons between the R_MKL model and all the 4 studied multi-omics models indicated that there were no significant differences in model discriminability between R_MKL and all other multi-omics models in the hold-out test set **(** **Figure 3D** and **Table 4B** **)**.

## Discussion

ART aims to compensate for patient-specific anatomic variations in NPC patients between fractions, while routine ART implementation on patient basis would undoubtedly pose immense burden to clinic. Previously, we were the first to demonstrate the capability of tumoral Radiomics from pre-treatment MRI for prediction of ART eligibility in NPC patients (15). In this study, we investigated a variety of single-omics and multi-omics models from multi-modal images, with an eye towards identifying their roles in predicting ART eligibility in NPC and providing insights into development of ART eligibility screening strategy in NPC in the long run. In this discussion, we attempted to highlight key findings of our study, scrutinize possible underlying reasons, and provide research community with potential directions in the future.

Results of our study showed that the R model significantly outperformed all other studied single-omics models (i.e., M, C and D models, all p-value < 0.0001), achieving an average AUC of 0.942 (STD: 0.009, 95%CI: 0.938-0.946) in the training set and 0.918 (STD: 0.034, 95%CI: 0.903-0.933) in the hold-out test set **(** **Figures 3A, B** and **Table 4A** **)**. Among the studied multi-omics models, the RMDC had the highest average AUC in both cohorts **(** **Figures 3C, D** and **Table 4B** **)**, however, its difference to the other three models (RM, RD and RC) did not reach the level of statistical significance in the hold-out test sets **(** **Table 4B** **)**. Surprisingly, there was no statistical difference between the R_MKL and all the studied multi-omics models in the hold-out set **(** **Table 4B** **)**. In other words, addition of other types of -omics features into a radiomic model did not demonstrate statistically significant improvement in model performance, suggesting the dominant role of Radiomic features in prediction of multifactorial ART eligibility in NPC. Besides, Radiomic features accounted for majority of the final selected features, ranging from 64% to 94%, in all the studied multi-omics models **(** **Table 3** **)**. We speculated that the dominant role of Radiomics found in this study could partially be explained by both the unique nature of Radiomics and the multi-factorial nature of the ART eligibility.

First, the outstanding predictability of Radiomics in this study may largely lie in its unique capability in unraveling intrinsic tissue property regarding response to treatment perturbations, which can be tissue-type dependent and patient-specific. There is mounting evidence in the literature showing the power of Radiomics in predicting treatment response in various cancer diseases (24–29). For instance, Hou et al. investigated CECT-based biomarkers for prediction of therapeutic response to chemo-radiotherapy in esophageal carcinoma and reported the discriminability of their model in AUC ranging from 0.686 to 0.727 (24). Wang et al. developed a radiomic signature combining features from multi-modal MR imaging sequences for prediction of early treatment response to induction chemotherapy in NPC patients, achieving an AUC of 0.822 (25). Piao et al. devised a MR-based radiomic model to distinguish sensitive and resistant tumors in NPC patients following induction chemotherapy, yielding an AUC of 0.905 (26). In these studies, the tumor response was defined in accordance with the Response Evaluation Criteria in Solid Tumors (RECIST) *via* quantitative assessment of tumor shrinkage, which follows the same line of thought as in this present study. Apart from this, Ramella et al. performed radiomic analysis of pre-treatment CT images of replanned non-small-cell lung cancer patients and generated a radiomic signature for prediction of tumor shrinkage during chemo-radiotherapy, yielding an AUC of 0.82 (27). Yu et al. analyzed tumoral radiomic features from multi-parametric pre-treatment MRI of NPC patients and developed several prediction models for ART eligibility, achieving AUC ranging from 0.750 to 0.930 (15). All the above evidence indicates the outstanding capability of Radiomics in divulging patient-specific intrinsic tissue biologic characteristics for discerning respondent and non-respondent cancer patients upon treatment perturbations, laying great foundation for predicting patient-specific anatomic variations for ART eligibility for NPC in this study.

By contrast, Dosimoics mainly characterizes aggressiveness of a specific treatment plan by capturing dose statistics from the entire three-dimensional dose distribution map within each of the studied organ structures, while it appears to convey little information on tissue responsiveness upon treatment perturbations. To a degree, this may shed some light on the well-recognized phenomenon where the same-staged patients experienced a diverse range of treatment outcome/response following identical treatment (same degree of treatment aggressiveness). Herein, we emphasize that results of our study do not deny the potential of Dosiomics in predicting treatment response. Indeed, it is worth noting that the D model was the second best-performing model in this study, giving rise to an average AUC of 0.895 (STD: 0.018, 95%CI: 0.887-0.903) in the training set and 0.811 (STD: 0.029, 95%CI: 0.798-0.824) in the hold-out test set **(** **Figures 3A, B** and **Table 4A** **)**. This result appears in agreement with most of the previous studies investigating triggering factors for ART in NPC (8–20), where radiation dose deposited was regarded as a prime factor for morphologic volume shrinkage of targets and OARs during the RT course, which may in turn incur intolerable dosimetric deviations from initial treatment plan and hence trigger ART implementation. For instance, Cheng et al. (9) analyzed repeated planning CT and MR scans at 30 and 50-Gy intervals. They reported that the shrinkage of both primary tumor and nodal lesions were higher when 50-Gy was delivered (13% and 29%, respectively) than that when 30-Gy was given (9% and 16%, respectively) and similar trend was also observed for bilateral parotid glands, which jointly led to significant increase in doses to numerous critical OARs, triggering implementation of ART. In this regard, several research groups have also suggested to incorporate dosimetric deviations in targets and/or OARs (such as parotid glands) as part of the ART regimen (12, 18–20). Of note, although Dosiomics has recently been studied for prediction of toxicity (32, 34, 41–43) and prognosis (33, 44) in cancer patients, its potential in treatment response prediction, in particular on the basis of the RECIST criteria, has not been reported. Future studies in this aspect are recommended to confirm its capability in this regard.

On the other hand, Morphologic and Contouromic features merely depict initial morphometric characteristics and geometric relationship between organs, respectively. They share commonality in their distinct disparity against Radiomics in that they both carry little or no underlying biologic information of the studied organ structures. This may in part explain the fair-to-poor predictive performance of the M and C models in our study, yielding an AUC of 0.643 (STD: 0.078, 95%CI: 0.608-0.677) and 0.550 (STD: 0.082, 95%CI: 0.514-0.582) in the hold-out test set, respectively **(** **Figures 3A, B** and **Table 4A** **)**.

In addition, the multifactorial nature of ART eligibility in the context of NPC disease may further elucidate why Radiomics plays a dominant role in this study, irrespective of additional types of -omics features. ART eligibility in NPC depends on multiple organs located in a confined space of head-and-neck regions. GTVnp, GTVn and bilateral parotid glands are all bulky organ structures within the nose-pharynx ministry, responsiveness of these structures upon treatment perturbations jointly determines the degree of patient-specific alternations in anatomy, hence affecting the demand for ART. Given the unique superiority of Radiomics in unravelling intrinsic tissue biologic response, we inferred that the role of Radiomics could become increasingly important when more organ structures come into play in contributing to the studied outcome (i.e., the ART eligibility), compared with other types of -omics features. This may, to some extent, provide an insight into our findings that Radiomic features accounted for the largest proportion of the final selected features in all the studied multi-omics models **(** **Table 3** **)**; and that the multi-organ-based R model performed far better than other single-omics models (all p-value < 0.0001) **(** **Table 4A** **)**; and that incorporating Morphologic and/or Dosiomic and/or Contouromic features into the radiomic model did not demonstrate statistically significant improvement in the hold-out test set **(** **Table 4B** **)** (all p-value > 0.05). Herein, we highlight that findings of this study may provide research community with valuable insights into development of pre-treatment stratification strategies for ART eligibility in NPC patients, potentially facilitating clinical implementation of ART in the future.

Although there exists a lack of studies on revealing multi-omics in prediction of multi-organ triggering outcome, results from a few studies in the literature may worth our attention. Sheikh et al. investigated radiomics and dosimetric features from bilateral parotid and submandibular glands (i.e., four separated organ structures) for predicting xerostomia, and reported that addition of dosimetric and clinical factors into a joint-CT-MR radiomic model did not lead to statistically significant improvement in model performance (45), which appears to be in line with our current findings. By contrast, Jiang et al. reported superior model performance when using both radiomic and dosimetric features from five lung sub-regions for predicting radiation pneumonitis than when using radiomic features alone (46), which may appear contradictive to our findings. However, it should be noted that the features in their studies were essentially derived from a single organ – the same lung tissue, rather than individual separated organ structures as in this current work. Further, unlike the present work, only CT-based radiomics was adopted in their study, which may lead to a relatively weaker predictive power than as if it were developed from multi-modal images that capture complementary tissue characteristics. Notwithstanding, this presents an interesting area to be explored and a close scrutinization of different types of features in prediction of a multi-organ contributing outcome is highly warranted in the future to further affirm the role of radiomics in context.

This study has several limitations. First, our models were developed and validated in a small-sized single cohort of NPC patients who received CCRT under Tomotherapy machine. While we believe such a homogeneous dataset is advantageous for model building, findings of our study require further validation in a large multi-cohort study. However, it is worth noting that the goal of this study was to assess the role of different omics-based prediction models for ART eligibility in NPC, instead of developing a generalizable model for clinical adoption. Thus, results of this study still deserve great attention in the community. Second, this study employed a large number of features for model building, which may lead to model overfitting in a small-sized cohort. In this regard, we deployed a strategic approach of determining optimal FS combinations that were used for feature dimensionality reduction prior to model development. The remnant feature sets were of high outcome relevance and low feature redundancy, and only 10 to 33 and 37 to 55 features were input to the modelling algorithms for developing single-omics and multi-omics models, respectively.

## Conclusion

Comparisons among all the studied models indicated that the Radiomic model was found to play a dominant role for ART eligibility in NPC patients; and Radiomic features accounted for the largest proportion of features in all the four multi-omics models, suggesting its governing power in ART eligibility prediction.

## Data Availability Statement

The patients’ clinical and DICOM data are not publicly available for patient privacy protection purposes. Requests to access these datasets should be directed to the corresponding author.

## Author Contributions

SL, YZ, JZ, BL, XT, ZM, TZ, TP, HX, TL, GR, AnC, FL, KA, VL, AmC, and LC contributed to study design, methodology development, results interpretation, and manuscript review. SL, JS, CL, BC, RN, FL, CY, and KA offered administrative and material support for clinical data and imaging data collection. SL and YZ constructed and validated the models. SL wrote the manuscript. JC supervised the study. All authors contributed to the article and approved the submitted version.

## Funding

This research was partly supported by research grants of Innovation and Technology Fund (ITS/080/19), the Innovation and Technology Commission, and Project of Strategic Importance Fund (P0035421), The Hong Kong Polytechnic University, The Government of the Hong Kong Special Administrative Region.

## Conflict of Interest

The authors declare that the research was conducted in the absence of any commercial or financial relationships that could be construed as a potential conflict of interest.

## Publisher’s Note

All claims expressed in this article are solely those of the authors and do not necessarily represent those of their affiliated organizations, or those of the publisher, the editors and the reviewers. Any product that may be evaluated in this article, or claim that may be made by its manufacturer, is not guaranteed or endorsed by the publisher.
