# Supplementary material for: Multi-Organ Omics-Based Prediction for Adaptive Radiation Therapy Eligibility in Nasopharyngeal Carcinoma Patients Undergoing Concurrent Chemoradiotherapy
Source: Front Oncol. 2022 Jan 31;11:792024. doi: 10.3389/fonc.2021.792024 (PMC8842229; doi:10.3389/fonc.2021.792024)
Supplement: Supplementary file 1 [file DataSheet_1.docx]

**Supplementary Material for**

**“Multi-organ Omics-based Prediction for Adaptive Radiation Therapy Eligibility in Nasopharyngeal Carcinoma Patients undergoing Concurrent Chemoradiotherapy”**

**Supplementary A1:**

**Image acquisition**

For CECT image acquisition, patients were immobilized with thermoplastic cast in a supine position. The scan range covered from the vertex to 5-cm below the sternoclavicular notch and was acquired at 3-mm intervals using a 16-slice Brilliance Big Bore CT (Philips Medical Systems, Cleveland, OH). Details of acquisition parameters: scan mode = Helical, voltage = 120-kVp, X-ray tube current = 264-mA, exposure = 325-msec, pixel spacing = 1.152x1.152-mm, slice thickness = 3-mm, matrix = 512x512 pixels. Two types of intravenous contrast agents were available: (i) OMNIPAQUE TM 350 mg I/ml and (ii) VISIPAQUE TM 320 mg I/ml; either one of them was prescribed and injected at a rate of 2-ml/sec for 70-ml, followed by scanning after a 30-sec delay.

MRI scans were acquired under a 1.5-Tesla MR scanner (Siemens Avanto, Germany). Details of MRI acquisition parameters: axial T2-weighted (T2-w) using short-tau-inversion- recovery (STIR) MR sequence (repetition time [TR]/ echo time [TE]: 7640/97-ms, field-of-view [FOV] = 24x24-cm^2^, number of acquisition = 1, slice thickness = 4-mm x 25 slices, spacing: 0.75x0.75x4.4-mm^3^, matrix: 320) and axial CET1-w spin-echo MR sequence TR/TE: 739/17-ms, FOV = 24x24-cm^2^, number of acquisition = 1, slice thickness = 3-mm x 48 slices, spacing: 0.938x0.938x3.3-mm^3^, matrix: 256).

**Supplementary A2:**

**Image pre-processing**

Prior to radiomic feature extraction, all CECT and MR images were resampled to a voxel size of 1x1x1-mm^3^ to mitigate impacts of difference in image acquisition parameters among different patients. Quantization of grey levels was applied to normalize image signal intensities in both types of images. Grey-level intensities of the images were discretized to a range of fixed bin counts, ranging from 50 to 350 with an incremental interval of 50. Besides, all images were convolved with Laplacian of Gaussian (LoG) filter under three levels of Gaussian radius parameters to produce filtered images which highlight specific texture radiomic features at multiple scales from fine (1 mm), medium (3 mm) to coarse (6 mm). Wavelet filters (HHH, HLL, LHL, LLH, LHH, HLH, HHL, LLL) were also applied to both CECT and MR images for yielding radiomic features with multiple resolutions. Re-segmentation was performed on CECT images to confine the Hounsfield Unit (HU) to the range of [-150, 180] for eliminating non-soft tissue components within the studied VOI, such as air cavities and bony structures. In particular to MR images, inhomogeneity correction of image pixel value was implemented using N4B bias correction provided in the “N4 Bias Field Correction Image Filter” in SimpleITK (v1.2.4).

**Supplementary A3:**

**Determination of optimal feature selection (FS) algorithms for each feature dataset**

**Supplementary Figure S1** outlines 24 combinations of the 4 supervised and 6 unsupervised FS algorithms studied. Each of the studied FS combinations was applied to training data of each iteration, followed by a 10-fold cross validation (CV) during Ridge modeling. More details of the studied FS algorithms can also be found in the literature (1-10).

**Supplementary Figure S2** illustrates a simplified workflow for optimal FS combination determination of a given feature set. The patient cohort was divided into a training and a hold-out dataset with 10 iterations. The hold-out test dataset of each iteration was employed to assess the model discriminability in terms AUC score to avoid introducing bias. Feature output stability of the models was assessed through the 10 iterations and quantified to a value ranging from 0 to 1 using a frequency-based criterion (11). A value of 1 for stability indicates that the model has completely consistent feature selection results under the 10 iterations. More details on the frequency-based criterion approach are explained as follows:

After implementing the workflow of **Supplementary Figure S2** with 10 iterations, a binary matrix Z which indicates results of the feature selection of each iteration can be obtained. The matrix Z is defined as follows:


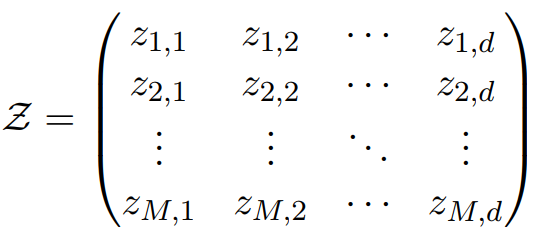


where each of the 10 rows represents results of the feature selection in each iteration. For example, z_1,d_=0 means the d^th^ feature was removed during the 1^st^ iteration. As such, feature output stability of the models was assessed through the 10 iterations and quantified to a value ranging from 0 to 1 using a frequency-based criterion, as adopted in a previous publication (11) and formulated as follows:


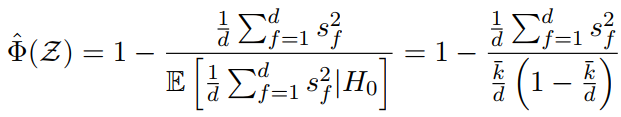


where


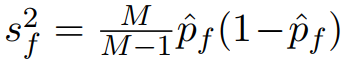


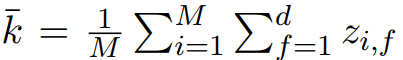


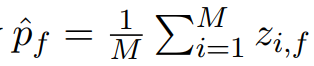


As such, scores of feature output stability (i.e., stability score) and model discriminability (i.e., average AUC score on the hold-out test datasets) for each FS combination can be determined for a particular feature dataset. In this study, a product of both scores was calculated and plotted in a decision graph **(Supplementary Figure S3)** for all the studied FS combinations. The FS combination with the highest value of the score product was determined to be the optimal FS combination for each of the studied feature datasets, which was used in subsequent model development.

**Supplementary A4:**

**MKL Algorithm**

MKL algorithm was applied for development of multi-omics models in this study. Unlike single-omics features, different types of multi-omics data may contain distinctly different data representations. In kernel learning, such data representations are implicitly selected through kernel. The use of kernel allows machine learning practitioners to define similarity between two types of -omics data and a proper regularization term for a learning task. Lanckriet et al. has demonstrated that adoption of multiple kernels improved model interpretability and classification performance when compared to single kernel application (12) and introduced MKL for binary classification (13).

From mathematical perspective, the MKL was adopted by reconstructing the kernel representation in Kernel Ridge Regression (KRR) to the following expression:

$$\mathbf{K}=\sum_{i=1}^{P} {\alpha_{i}\mathbf{K}}_{i}$$

where K_i represents different kernel matrices constructed by different types of kernels, α_i is the kernel weight of K_i, P is the number of kernel matrices. More information on KRR can be found in a previous literature (14).

**Supplementary Figure S4** illuminates the multi-omics fusion framework in our study. Considering a multi-omics feature dataset containing “s” types of -omics data and a total number of “t” features, M_1_, M_2_, M_s_ refer to all the features in the 1^st^, 2^nd^ and all types of -omics data, respectively; f_1_, f_2_, f_t_ refer to the 1^st^, 2^nd^, t^th^ individual features of the given multi-omics feature dataset. KM refers to kernel matrix. Gaussian [0.5, 1, 2, 5, 7, 10, 12, 15, 17, 20] corresponds to kernel parameters used in the Gaussian kernel function; Polynomial [1, 2, 3] corresponds to kernel parameters used in the Polynomial kernel function. As such, each of the kernel matrices (KM) can either use the combined set of multi-omics features or individual set of single-omics features under specific kernel parameters, achieving multi-omics fusion.

**References:**

1. Wright S. The Interpretation of Population Structure by F-Statistics with Special Regard to Systems of Mating. *Evolution* (1965) 19(3):395-420. doi: 10.2307/2406450.
2. John C.D. Statistics and Data Analysis in Geology (3^rd^ edition). Wiley (2002).
3. Duda R.O., Hart P.E., Stork D.G. “Pattern Classification”. New York: John Wiley & Sons (2007), 24: 305-7.
4. Robnik-Šikonja M, Kononenko I. Theoretical and Empirical Analysis of ReliefF and RReliefF. *Mach Learn* (2003) 53(1/2):23-69. doi: 10.1023/a:1025667309714.
5. He, X., Cai, D., & Niyogi, P. Laplacian Score for Feature Selection. NIPS (2005).
6. Zheng Z., Liu H. Spectral Feature Selection for Supervised and Unsupervised Learning. In Proceedings of the 24th international conference on Machine learning (ICML '07). Association for Computing Machinery (2007), New York, NY, USA, 1151–1157. DOI:https://doi.org/10.1145/1273496.1273641
7. Cai D., Zhang C., He X. Unsupervised feature selection for multi-cluster data. In Proceedings of the 16th ACM SIGKDD international conference on Knowledge discovery and data mining (KDD '10). Association for Computing Machinery (2010), New York, NY, USA, 333–342. DOI:https://doi.org/10.1145/1835804.1835848
8. Li Z., Tang J. Unsupervised Feature Selection via Nonnegative Spectral Analysis and Redundancy Control. IEEE Transactions on Image Processing (2015)24(12):5343-55. doi: 10.1109/tip.2015.2479560.
9. Yang, Y., Shen, H.T., Ma, Z., Huang, Z., & Zhou, X. L2, 1-Norm Regularized Discriminative Feature Selection for Unsupervised Learning. IJCAI (2011).
10. Tadist K., Najah S., Nikolov N.S., Mrabti F., Zahi A.. Feature Selection Methods and Genomic Big Data: A Systematic Review. *J Big Data* (2019) 6(1). doi: 10.1186/s40537-019-0241-0.
11. Sechidis K., Papangelou K., Nogueira S., Weatherall J., Brown G. On the Stability of Feature Selection in the Presence of Feature Correlations. In: Brefeld U., Fromont E., Hotho A., Knobbe A., Maathuis M., Robardet C. (eds) Machine Learning and Knowledge Discovery in Databases (2020). ECML PKDD 2019. Lecture Notes in Computer Science, vol 11906. Springer, Cham. https://doi.org/10.1007/978-3-030-46150-8_20.
12. Lanckriet G.R.G., De Bie T., Cristianini N., Jordan M.I., Noble W.S. A Statistical Framework for Genomic Data Fusion. *Bioinformatics* (2004) 20(16):2626-35. doi: 10.1093/bioinformatics/bth294.
13. Lanckriet G.R.G., Cristianini N., Ghaoui L.E.l., Bartlett P., Jordan M.I. Learning the Kernel Matrix with Semi-definite Programming. Technical Report (2002). University of California at Berkeley, USA.
14. Deng Z., Choi K.S., Jiang Y., Wang S. Generalized Hidden-Mapping Ridge Regression, Knowledge-Leveraged Inductive Transfer Learning for Neural Networks, Fuzzy Systems and Kernel Methods. IEEE Transactions on Cybernetics (2014) 44(12):2585-99. doi: 10.1109/tcyb.2014.2311014

**Supplementary Figure S1**


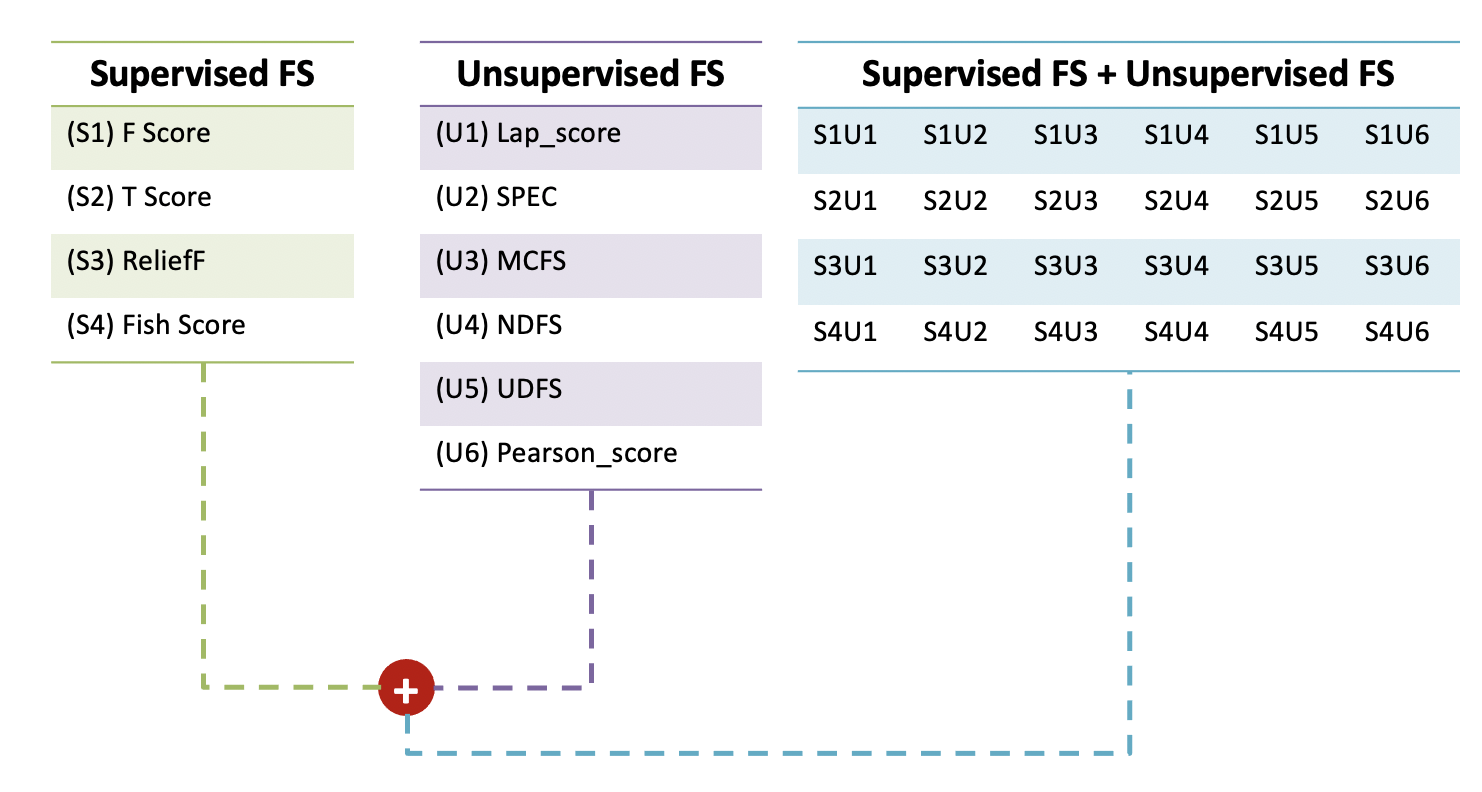


**Figure S1.** A summary of all the studied supervised and unsupervised FS algorithms and the studied FS combinations. Abbreviations: Lap score: Laplacian score, SPEC: Spectral, MCFS: multi-cluster feature selection, NDFS: Nonnegative discriminative feature selection, UDFS, Unsupervised discriminative feature selection.

**Supplementary Figure S2**

**Figure S2.** A simplified workflow for optimal FS combination determination of a given feature set.

**
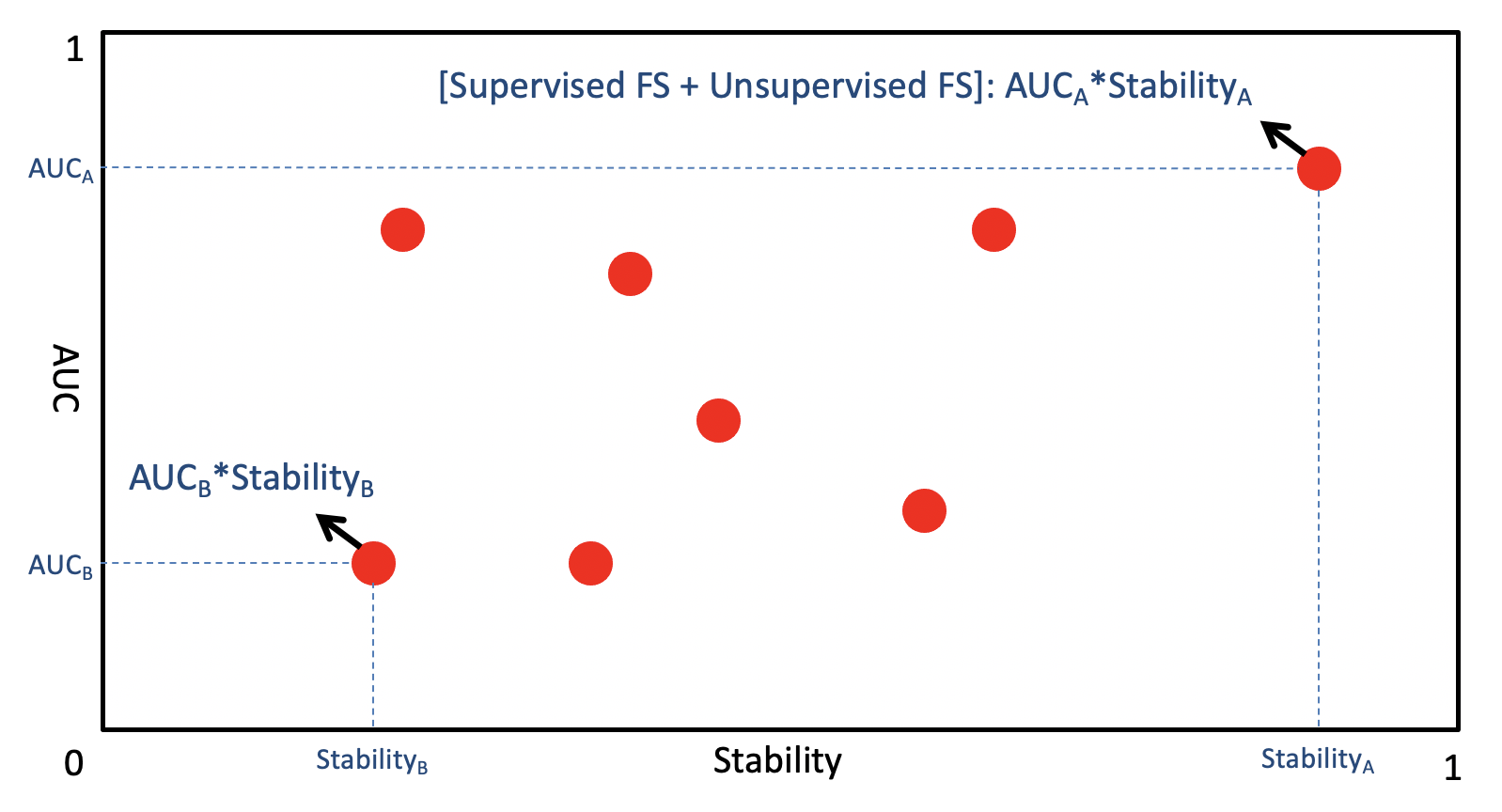
**

**Figure S3.** A decision graph showing results of the product of stability score and average AUC score for 8 different FS combinations of a given feature set. Point A has the highest product score of stability and AUC, and hence refers to the optimal FS combination while Point B represents the worst FS combination.

**Figure S4.** Schematic diagram of the multi-omics fusion framework. Considering a multi-omics feature dataset containing “s” types of -omics data and a total number of “t” features, M_1_, M_2_, M_s_ refer to all the features in the 1^st^, 2^nd^ and all types of -omics data, respectively; f_1_, f_2_, f_t_ refer to the 1^st^, 2^nd^, t^th^ individual features of the given multi-omics feature dataset. KM refers to kernel matrix. Gaussian [0.5, 1, 2, 5, 7, 10, 12, 15, 17, 20] corresponds to kernel parameters used in the Gaussian kernel function; Polynomial [1, 2, 3] corresponds to kernel parameters used in the Polynomial kernel function.

**
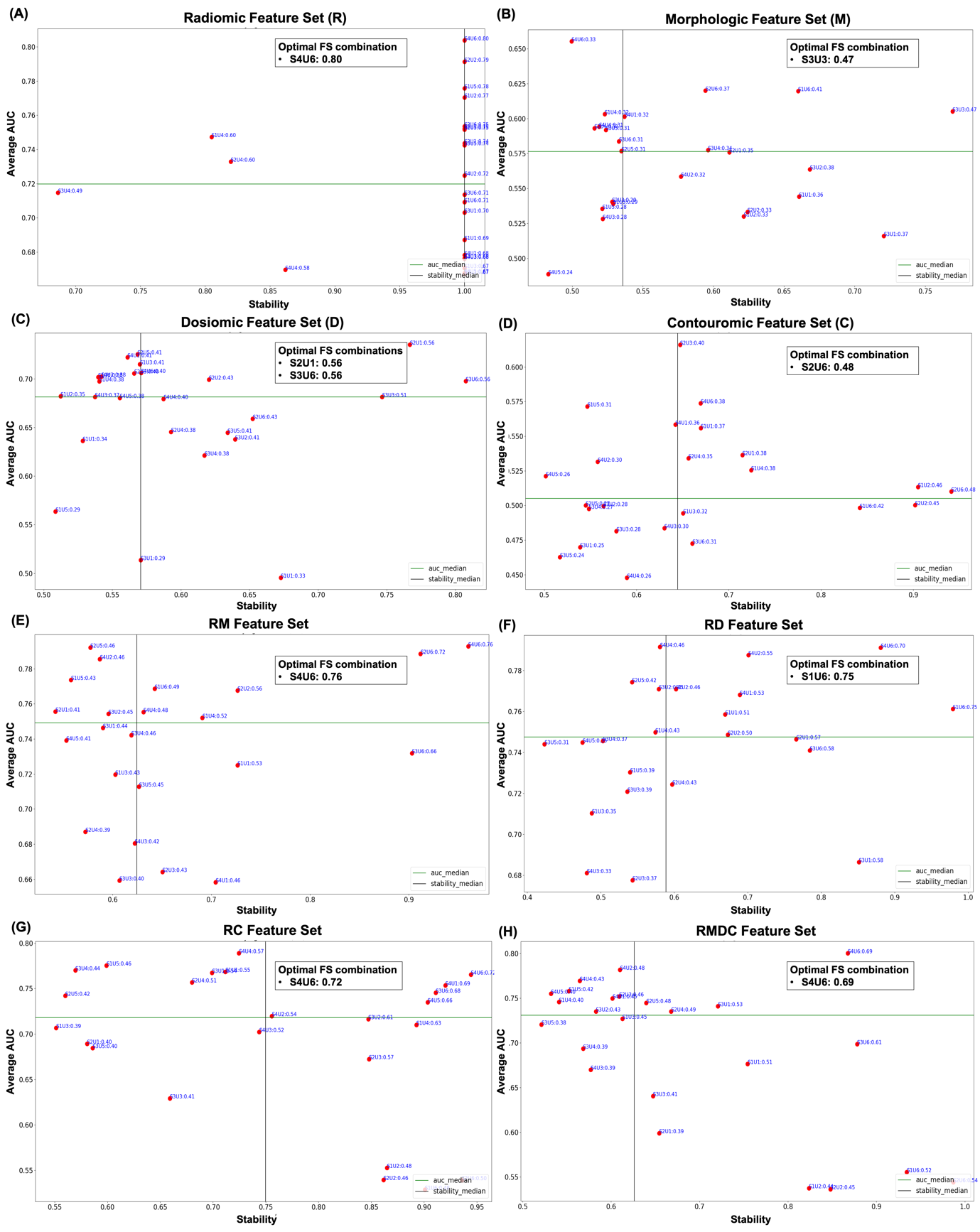
**

**Figure S5(A-H).** Results of decision graphs for the four single-omics feature sets (A-D) and the four multi-omics sets (E-H), with the corresponding optimal FS algorithms and the product scores indicated inside the box of each graph.

**
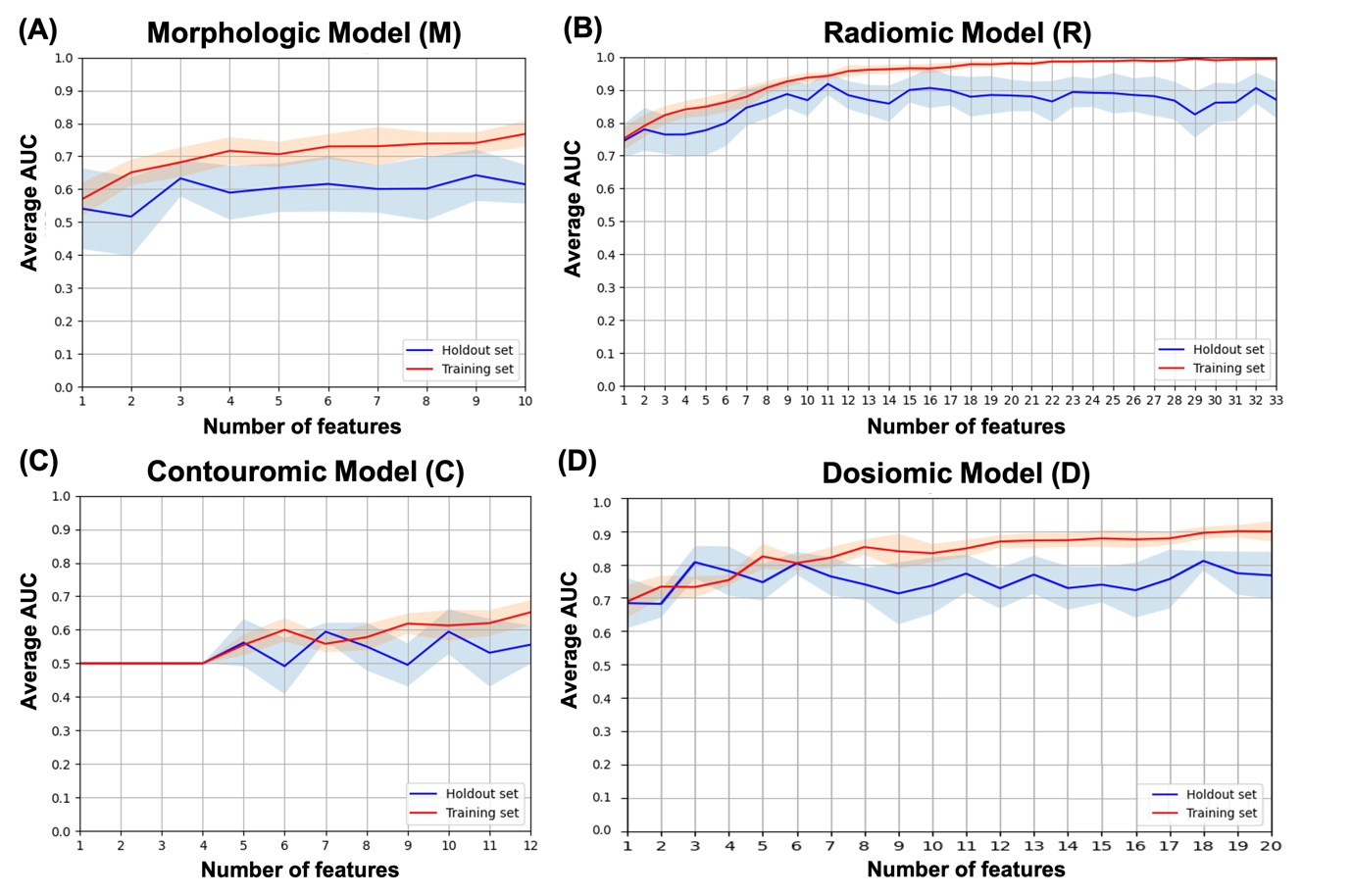
**

**Figure S6(A-D).** Change of average AUC scores (and its STD shown in shadow) in both training (red curves) and hold-out test sets (blue curves) against varying number of features for the four single-omics models: Morphology (A), Radiomics (B), Contouromics (C), and Dosiomics (D), respectively.


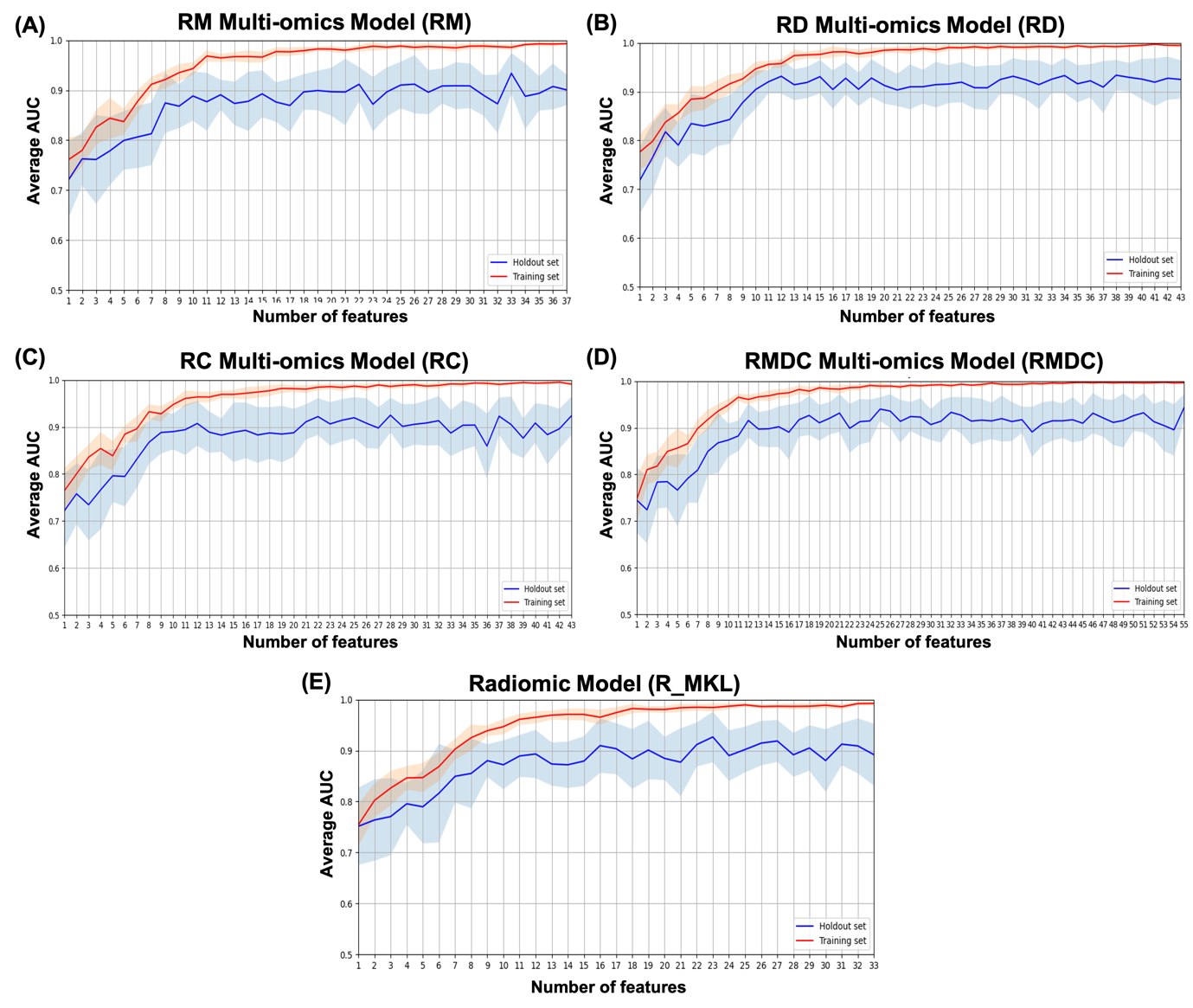


**Figure S7(A-D).** Change of average AUC scores (and its STD shown in shadow) in both training (red curves) and hold-out test sets (blue curves) against varying number of features for the four multi-omics models: Radiomics+Morphology (A), Radiomics+Dosiomics (B), Radiomics+Contouromics (C), and Radiomics+Morphology+Dosiomics+Contouromics (D), and the Radiomics models trained by using MKL algorithm (E), respectively.

**Table S1.** Results of optimal combinations of supervised and unsupervised feature selection (FS) algorithms for the four single-omics models and four multi-omics models.

| **Feature Set** | **Optimal FS Combination** |
| --- | --- |
| **Single-omics Model** | |
| Radiomics (R) | S4U6 |
| Morphology (M) | S3U3 |
| Dosiomics (D) | S2U1, S3U6 |
| Contouromics (C) | S2U6 |
| **Multi-omics Model** | |
| RM | S4U6 |
| RD | S1U6 |
| RC | S4U6 |
| RMDC | S4U6 |
